# Micronutrients and in vivo antioxidant properties of powder fractions and ethanolic extract of *Dichrostachys glomerata* Forssk. fruits

**DOI:** 10.1002/fsn3.1606

**Published:** 2020-05-14

**Authors:** Markusse Deli, Elie Djantou Baudelaire, Richard Marcel Nguimbou, Nicolas Njintang Yanou, Joël Scher

**Affiliations:** ^1^ Food Sciences and Nutrition ENSAI University of Ngaoundere Ngaoundere Cameroon; ^2^ Department of Biological Sciences Faculty of Sciences University of Ngaoundere Ngaoundere Cameroon; ^3^ Laboratoire d'Ingénierie des Biomolécules (LIBio) Université de Lorraine Vandœuvre‐lès‐Nancy France

**Keywords:** antioxidant, *Dichrostachys glomerata* fruits, ethanol extraction, minerals, powder, sieved fractionation, vitamins

## Abstract

*Dichrostachys glomerata* powders were processed by sieve fractionation and ethanolic extraction followed by freeze‐drying. The micronutrient contents and the in vivo antioxidant properties of powder fractions in rats' high‐fat diet‐induced oxidation model were determined. Sieved fractionation was achieved by finely grinding the Dichrostachys fruits and fractionating on a sieve column to retain particle of sizes <180, 180–212, 212–315, and ≥315 µm. Unsieved powder and lyophilized ethanolic extract were used as control. All the powders were examined for the minerals, total carotenoids, and vitamins contents. For in vivo antioxidant properties assessment, the individual powder was dissolved in water and administered to rats at the dose of 250 mg/kg body weight. Oxidation was induced by treating the rat with high‐fat diet, and the measured parameters were malondialdehyde, superoxide dismutase, and catalase activities. The results showed a significant influence (*p* < .05) of particle size on the micronutrient contents and in vivo antioxidant properties. The smaller the particle size of the powder fractions, the higher the minerals, vitamins, total carotenoids contents, and antioxidant properties. Comparatively, the ethanolic powder had the highest carotenoids content, while the powders of particle size <180 µm and 180–212 µm had the highest minerals and vitamin contents. The highest antioxidant properties were characterized by high superoxide dismutase, catalase activities, and low malondialdehyde production. The grinding of Dichrostachys fruit followed by controlled differential sieving process may compete with ethanol extraction for an efficient concentration of bioactive compounds and micronutrients except carotenoids.

## Graphical abstract

Dichrostachys glomerata powders were processed by sieve fractionation and ethanolic extraction followed by freeze‐drying. The micronutrients contents and the in vivo antioxidant properties of powder fractions in rats' high‐fat diet‐induced oxidation model were determined. The grinding of Dichrostachys fruit followed by controlled differential sieving process may compete with ethanol extraction for an efficient concentration of micronutrients and antioxidant bioactive compounds.

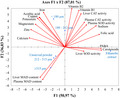

## 1 INTRODUCTION

Spices produce an extremely large range of natural products and essential micronutrients which can be responsible for their therapeutic effect. Specifically, plant‐derived substances such as phenolic compounds, vitamins, carotenoids, and some minerals have been reported to have a great scientific interest and well‐established biological effects. Of paramount importance, antioxidant activity is supposed to be the basis of numerous bioactivities in plants. Indeed, natural antioxidants contained in edible plants are often believed to play an important role in inhibiting oxidation process by quenching free radicals, chelating catalytic metal, and scanvenging oxygen in foods and biological systems. Therefore, *Dichrostachys glomerata* fruits is a plant example that has been largely reported to enclose many bioactive molecules such as phenolic compounds, vitamins, minerals and numerous medical, nutraceutical, and biological effects including antioxidant properties (Abdou Bouba et al., 2012; Abdou Bouba, Njintang, Scher, & Mbofung, 2010; Deli, Baudelaire, et al., 2019).

Although bioactive compounds may be accumulated in some parts of plants, they can only be found at very low levels to exert an optimal biological activity. Thus, extraction represents an essential operation to concentrate active compounds, since it ensures separation of this compounds from plant cellular matrix. The extraction conditions determine the quality and quantity of extracted active compounds (Ameer, Shahbaz, & Kwon, 2017; Shahbaz, Park, Kim, Akram, & Kwon, 2016). Plant extract is commonly obtained by using standard solvent methods with particular performances, benefits, and limits. Generally, ethanol and water are commonly used for extraction of plant active compounds as they have been classified an recognized as safe solvents (Monroy, Rodriguesb, Sartorattob, & Cabral, 2016). Unfortunately, all the standard solvent methods have been associated with high requirements such as long exposure time with the risk to damage oxidable constituents and results in low yields of extraction (Azmir et al., 2013). Standard methods also involved environment and toxicological requirement to reduce quantity of organic solvents used in the extraction process (Baudelaire, 2013; Palmade‐Le Dantec & Picot, 2010). These limitations have raised some criticisms from consumers and industries and open the way in developing new and alternative methods with significant advantages as compared to standard extraction with solvents.

Alternation of drying and grinding (ADG), and controlled differential sieving process (CDSp), is an effective method for efficient concentration of plant active ingredients. In this method, the plants materials are dried, finely grinded, and fractionated in a column of sieves with decreasing sizes. To date, numerous studies on controlled differential sieving of plant products have been published (Baudelaire, 2013; Becker et al., 2016, 2017; Deli, Baudelaire, et al., 2019; Deli, Petit, et al., 2019; Zaiter et al., 2017). Generally, these authors reported that concentration of bioactive compounds enhances the biological properties. However, effectiveness of ADG followed by CDSp as compared to standard solvent extraction methods has not been clarified. Nevertheless, Soualeh, Stiévenard, Baudelaire, Soulimani, and Bouayed (2018) and Soualeh, Stiévenard, Baudelaire, Bouayed, and Soulimani (2019) reported the cytoprotective and antioxidant activities of powder fractions of *Rosa canina* and *Salix alba* as compared to hydroethanolic extraction. According to these authors, better antioxidant and cytoprotective effects were found with superfine powders (i.e., 50–100 µm and 100–180 µm, both) of *S. alba* and intermediate fraction powder (i.e., 100–180 µm) of Rosa canina than an hydroethanolic extraction. Otherwise, our previous work on *D. glomerata* fruit powders demonstrated that granulometric fraction powder of 180–212 µm was found to maximize phenolic compound contents and in vitro antioxidant activity (Deli, Baudelaire, et al., 2019). Therefore, the present work aims to assess the effect of powder particle sizes on mineral, vitamins contents, and in vivo antioxidant properties of *D. glomerata* compared to ethanol extraction.

## 2 MATERIALS AND METHODS

### 2.1 Reagents, standards, and plant sample

All the chemicals and solvents were of standard analytical purity and purchased from Sigma‐Aldrich. Dried *D. glomerata* Forssk. fruits were obtained from local markets. Samples were hand‐cleaned of foreign bodies (inorganic materials, dirt, and dust particles) before been ground for powder production.

### 2.2 Methods

#### 2.2.1 Plant grinding and controlled differential sieving process

Grinding was operated at 8,049.6 *g* using an electric Ultra‐Centrifugal Mill ZM 200 supplied with 24‐tooth rotor of 99 mm and trapezoid holes mesh sieve of 1 mm. Obtained powder was sieved according to procedure previously mentioned by Deli, Baudelaire, et al. (2019) and Deli, Petit, et al. (2019). For that, 100 g of powder passed through sieve columns using an Analysette 3 Spartan apparatus (Fritsch) to obtain fraction powders. Sieve shaker vibration amplitude was set at 0.5 mm for 10 min. Thus, powder samples were classified into four main grades as follows: <180, 180–212, 212–315, and ≥315 µm. Unsieved powder was taken as control, and they were packed in plastic bags and stored at 10°C until analysis.

#### 2.2.2 Ethanol extraction and production of ethanol fraction

Unsieved powder was mixed with ethanol in the ratio of 1/10 (w/v) and stand under magnetic stirring (Variomag Poly) for 24 hr at 18°C. Then, mixture was filtered using a Waltman paper of pore size 12–15 µm. Ethanol was removed using a rotary evaporator (BUCHI ‐ R210/215) at 40°C and under reduced pressure of 175 mbar. Obtained concentrate extract was kept in a freezer at −18°C for 24 hr, and finally, frozen extract was next freeze‐dried at −60°C for 48 hr. Freeze‐dried collected extract was conditioned in plastic bags and stored at 10°C until analysis.

#### 2.2.3 Determination of micronutrients contents

##### Determination of minerals contents

Minerals were analyzed on ash samples obtained by incineration of powder samples in a muffle furnace at 550°C. Ash was then dissolved in 10% HCl solution prepared in deionized water (Oshodi, 1992). For quantification of calcium and magnesium, ash was dissolved in 10 ml of lanthanum chloride solution. All the solutions were filtered on Whatman No. 1 filter paper. Sodium and potassium were quantified by flame photometer, while atomic absorption spectrometer (Thermo Scientific, ICE 3000, and version 2.0) was used to quantify calcium, magnesium, zinc, iron, and copper. Suitable salts of metals were used to prepare standards, and specific lamps were fixed. Standard solutions of minerals at various concentrations were injected to calibrate the atomic absorption spectrometer using acetylene gas: K and Na (2–8 µg/ml), Ca (2–10 µg/ml), Mg (0.5–3 µg/ml), Fe (2.5–20 µg/ml), Zn (0.2–2 µg/ml), and Cu (0.5–4 µg/ml). Then, an aliquot of ash solutions was injected and their concentrations were obtained from standard curves.

##### Carotenoid extraction and determination

The method described by Rodriguez‐Amaya (2001) was used for determination of total carotenoids. 2.5 g of powder and 25 ml acetone were added successively to tube, and 75 mg of magnesium carbonate was added. Mixture was stand under constant magnetic stirring (Variomag Poly) for 10 min at 18 ± 2°C. Mixture was centrifuged (Thermo Scientific, Heraeus Megafuge 8R Centrifuge) at 603 g for 10 min at 10°C. Organic phase was collected, and extraction was repeated on residue with 5 ml of acetone and 5 ml of petroleum ether, followed by centrifugation. This procedure was repeated three times until the sample became colorless. Collected solutions were combined and transferred into a 500‐ml separation funnel containing 20 ml of petroleum ether. Acetone was removed through the slow addition of 150 ml of distiller water to prevent emulsion formation, and aqueous phase was discarded. Then, obtained extract was transferred through a funnel containing 15 g of anhydrous sodium sulfate and made up a volume of 25 ml with petroleum ether. Finally, absorbance was read on a UV spectrophotometer (Shimadzu UV‐VIS 1605) at 450 nm, and total carotenoid contents expressed in microgram per 100 gram of dry weight were calculated as follows:

$$\text{Total}\;\text{carotenoid}\;\text{content}\;\left(\mu g/100\;g\;\text{DW}\right)=\frac{\left(A\text{.V}.\;1,000\right)}{A_{1\;\text{cm}}^{1\%}\times w}\times 100$$

where Abs: absorbance at 450 nm, *V*: used volume of powder extract (ml), *w*: sample weight (g), and $A_{1\;\text{cm}}^{1\%}$ = 2,592 (β‐carotene extinction coefficient in petroleun ether).

##### Determination of some vitamin contents

Samples were prepared and analyzed using a validated method of simultaneous determination of water‐soluble and fat‐soluble vitamins (Kucukkolbasi, Onur, Ayyildiz, & Kara, 2013).

###### Standards and samples preparation

0.1 g of powder was extracted with mixed solvents of ratio 80:20 (v/v) (0.01% trichloroacetic acid prepared in ultrapure water and 100% methanol) under magnetic stirrer for 30 min and not exposed to bright light. Obtained solution was centrifuged at 603 *g* for 15 min at 4°C and filtered through a nylon syringe filter (pore size 0.45 µm).

Vitamin standards were dissolved in ultrapure water and methanol, respectively, for water‐soluble vitamins (thiamin, riboflavin, ascorbic acid, folic acid, and p‐aminobenzoic acid) and fat‐soluble vitamins (retinol acetate and tocopherol). Then, mixed solutions of vitamin standards at varied concentrations were prepared in water‐methanol mixture (50:50, v/v).

###### High‐performance liquid chromatographic (HPLC) analysis

High‐performance liquid chromatographic system equipped with a water pump, an automatic injectors, a UV/photodiode array detector (PDA), and C18 column (150 mm × 2.1 mm, 5 µm; AlltimaTM, serial number: 212120004) at 30°C was used. Mobile phase was a mixture of A (0.01% trichloroacetic acid of pH 4) and B (100% methanol) of grade HPLC, and 0.2 ml/min flow rate with a gradient run. A linear gradient program was used: 0–4 min, 95% A; 4–10 min, 95% B; 10–30 min 95% B; and 30–55 min 95% A. Five microliter of sample solution or vitamin standard was eluted. The HPLC peaks of vitamins were identified and quantified by comparing sample peak retention time. Calibration curves were prepared using series of vitamins standard solutions, and applied method was validated and given acceptable values in the regression coefficients (*R* ^2^) of calibration curves ranged between 0.986 and 0.999. Wavelengths used for determination of vitamin contents were as follows: 318, 292, 246, 246, 268, 290, and 288 nm of retinol acetate, α‐tocopherol, ascorbic acid, thiamin, riboflavin, folic acid, and p‐aminobenzoic acid, respectively. Results were expressed as microgram of vitamin standard equivalent per gram dry weight.

#### 2.2.4 Evaluation of in vivo antioxidant activity

##### Animals and experimental design

Adult male Wistar rats (*Rattus norvegicus*) were used for the experiments. There were raised at the animal house of Laboratory of Biophysics and Food Biochemistry and Nutrition (LABBAN) of the National School of Agro‐Industrial Sciences, Ngaoundere University of Cameroon. At 3 months old, rats weighting 200–300 g were randomized into eight groups (5 per cage) with ad libitium access to water and formulated normal and hyperlipidic diets (Table 1). A high‐fat diet (or hyperlipidic diet) was chosen as a generator of oxidant stress in the bodies of rats, as described by Ngatchic, Njintang, Oben, and Mbofung (2013). Oxidative stress generated by high‐fat diet induces an excessive production of free radicals. When the ROS is excessive, the homeostasis will be disturbed, resulting in oxidative stress (Rehman & Akash, 2017). Basic constituents of formulated high‐fat diet were represented by egg yolk (100 g of egg yolk can provide about 1.14 g of cholesterol) and coconut oil chosen for high saturated fatty acid contents (65%, w/w). As treatment, 10 ml aqueous solution of each fraction powders of *D. glomerata* at 250 mg/kg body weight or ascorbic acid at 20 mg/kg body weight was orally administrated for 28 consecutive days. Groups were organized as follows:

Normal control group: Rats received normal diet and distilled water for 28 days.
Negative control group: Rats received hyperlipidic diet and distilled water for 28 days.
Positive control group: Rats received hyperlipidic diet and were treated with 20 mg/kg body weight of ascorbic acid for 28 days.
Test groups (6): Rats received hyperlipidic diet and 10 ml of each powder solution from *D. glomerata* (fractions powders of <180, 180–212, 212–315, and ≥315 µm, unsieved powder and lyophilized ethanolic extract) at 250 mg/kg body weight. Rats groups administrated powder solutions were prepared by maceration of plant powder in water and stand under magnetic stirring for 2 hr.

**TABLE 1:** Composition of normal and hyperlipidic diets (Ngatchic et al., 2013)

Constituents	Ingredients	Normal diet		Hyperlipidic diet	
		Contents (g/kg)	Nutritional energy (kJ)	Contents (g/kg)	Nutritional energy (kJ)
Proteins	Fish powders	250	3,347.2	100	2,343.04
Carbohydrates	Corn starch	590	9,874.24	190	4,736.29
	Sugar	50	836.8	50	836.8
Lipids	Coconut oil	0	0	250	2,250
	Egg yolk	0	0	300	11,296.8
	Soya oil	50	1,882.8	50	1,882.8
Others	Cellulose	50	0	0	0
	Minerals (bone powder)	50	0	50	0
	Vitamin B complex	10	0	10	0
Total		1,000	15,941.04	1,000	30,928.128

###### Plasma and liver homogenate preparation

At the end of the experience period, the rats were fasted overnight and anesthetized to obtain 1–2 ml of blood sample drawn by cardiac puncture, and this blood was centrifuged at 603 *g* for 10 min to obtain the serum which was kept frozen at −4°C until used for analysis. Liver was equally cutted from three rats and liver homogenate prepared for determination of oxidant stress parameters. Protein content in liver homogenate and blood plasma was measured according to the method of Lowry, Rosebrough, Farr, and Randall (1951).

##### Determination of lipid peroxidation

Lipid peroxidation was estimated spectrophotometrically as described by Yagi (1976). In this respect, 100 μl of homogenate liver or blood plasma, 400 μl of TBA reagent, and 80 μl of HCl were successively introduced into a test tube. Mixture was vortexed and incubated in a boiling water bath for 15 min. After cooling in a cold water bath for 30 min, mixture was centrifuged at 603 *g* for 15 min. Absorbance of collected supernatant was read at 530 nm using UV‐visible spectrophotometer. Results was expressed as malondialdehyde (MDA) content in μmol/mg protein using molar extinction coefficient of MDA (*ε* = 1.56, 105 mol^−1^.cm^−1^).

##### Measurement of superoxide dismutase and catalase activities

Superoxide dismutase (SOD) activity was detected according to the method of Beauchamp and Fridovich (1971). 2.5 ml of 0.1 M carbonate buffer solution at pH 10.2 was added to 0.2 ml of liver homogenate or blood plasma. Then, 0.3 ml of adrenalin solution prepared at 5 μg/ml in water was added to the mixture to trigger the reaction and whole was vortexed. Absorbance was measured every 30 s until 150 s in order to follow an increase of absorbance at 480 nm. 0.3 ml of distilled water was used in a reference tube. Calculated SOD activity was expressed as units per milligram of protein.

Catalase (CAT) activity was detected as describe method of Sinha (1972). One milliliter of 0.1 M phosphate buffer at pH 7.4 and 0.4 ml of 0.2 M H_2_O_2_ were added to 100 μl of liver homogenate or blood plasma contained in tube. The reaction was stopped at 30, 60 and 90 s by adding 2 ml of dichromate/acetic acid mixture (5:95, v/v). Absorbance was measured at 620 nm, and CAT activity expressed in units per milligram of protein using molar extinction coefficient of CAT (*ε* = 0.036 mmol^−1^.cm^−1^).

#### 2.2.5 Statistical analysis

Obtained data were recorded in Excel file, and analysis was carried out in triplicate. Results were expressed as mean ± standard error mean deviation. One‐way analysis of variance (ANOVA), followed by Duncan's multiple range test, performed by Statgraphics was used to determine significant differences (*p* ≤ .05) among the samples. Principal components analysis (PCA) was performed using XLSTAT to highlight correlation between studied samples and minerals, vitamins, and carotenoid contents.

## 3 RESULTS AND DISCUSSION

### 3.1 Minerals contents

Minerals contents of *D. glomerata* powdered fractions are summarized in Table 2. Unsieved powder of *D. glomerata* contained considerable amounts of potassium, followed by magnesium, calcium, and iron (1,203.0, 137.3, 209.8, and 25.8 mg/100 g DW, respectively), while sodium (8.2 mg/100 g DW), zinc (1.43 mg/100 g DW), and copper (0.54 mg/100 g DW) contents were the lowest. Previous studies by Abdou Bouba et al. (2012) equally reported similar order of magnitude, but their potassium level was three times lower than ours. They equally reported significant level of selenium (110 mg/100 g DW), a variable which was not possible to detected under our working condition. Looking to the powder fractions, mineral contents significantly (*p* < .05) varied from one treatment to another according to the particle size. Generally, the highest contents of minerals were observed in the smallest particle size (powders of <180 µm). Indeed, mineral content was increased with decrease in particle size. This result supports the earlier reported hypothesis by several authors (Becker et al., 2016; Deli, Baudelaire, et al., 2019; Deli, Petit, et al., 2019; Flávia, Edwil Aparecida De, & Fernando, 2016; Lucas‐González, Viuda‐Martos, Pérez‐Álvarez, & Fernández‐López, 2017) and confirmed that finest powders from grinded and sieved vegetable matrix would more concentrate minerals than that of larger particle size. Indeed, these authors reported the highest ash contents in the finest particles size as compared to that of larger particles size. Thus, higher total ash content of finer particle powders supposed that their mineral contents are relatively higher. Interestingly, CDSp has advantage to concentrate more minerals as compared to lyophilized ethanolic extract. The results bring out the interest of CDSp to enhance the mineral content in plant extracts. Minerals such as copper, zinc, and iron are not antioxidants as such, but act as cofactors to maintain the catalytic activity of antioxidant enzymes (superoxide dismutase and catalase). It may therefore be hypothesized that finer powders from CDSp may exhibit higher antioxidant properties as compared to powders with larger particle size and powder from ethanol extract.

**TABLE 2:** Some minerals, vitamins, and total carotenoid contents of powder fractions obtained by CDSp, unsieved powder, and freeze‐dried ethanolic extract from *D. glomerata* fruits

Constituents	Powder samples from *Dichrostachys glomerata* fruits					
	<180 µm	180–212 µm	212–315 µm	≥315 µm	Unsieved powder	Lyophilized ethanolic extract
Minerals (mg/100 g DW)						
Potassium	1,800 ± 9^f^	1,527 ± 1^e^	1,228 ± 6^d^	1,079 ± 15^b^	1,203 ± 12^c^	522.9 ± 26.1^a^
Calcium	174.1 ± 8.2^c^	141.0 ± 7.2^b^	171,9 ± 2.6^c^	140.1 ± 2.1^b^	137.3 ± 6.8^b^	19.42 ± 0.32^a^
Magnesium	363.3 ± 13.9^d^	203.6 ± 7.1^bc^	189.3 ± 6.6^b^	212.7 ± 11.9^c^	209.8 ± 8.1^c^	42.91 ± 0.35^a^
Sodium	11.47 ± 0.02^e^	11.15 ± 0.03^d^	8.89 ± 0.03^c^	5.52 ± 0.04^a^	8.23 ± 0.02^b^	15.59 ± 0.23^f^
Iron	46.54 ± 0.19^f^	34.85 ± 0.04^e^	24.84 ± 0.13^c^	18.66 ± 0.10^b^	25.83 ± 0.01^d^	10.32 ± 0.06^a^
Zinc	1.93 ± 0.01^d^	1,57 ± 0.03^c^	1.43 ± 0.01^b^	1.44 ± 0.01^b^	1.43 ± 0.01^b^	0.32 ± 0.01^a^
Copper	0.86 ± 0.01^f^	0.63 ± 0.01^e^	0.58 ± 0.01^d^	0.52 ± 0.03^b^	0.54 ± 0.01^c^	0.32 ± 0.01^a^
Vitamins (µg/100 g DW)						
Ascorbic acid*	69.00 ± 5.42^c^	67.12 ± 5.42^c^	61.07 ± 1.57^b^	60.67 ± 4.26^b^	59.72 ± 1.57^b^	52.72 ± 0.27^a^
Thiamin	13.01 ± 0.80^b^	12.96 ± 0.74^b^	12.35 ± 0.70^ab^	11.51 ± 0.53^a^	12.23 ± 0.83^ab^	12.77 ± 0.10^ab^
Riboflavin	328.1 ± 16.3^c^	314.1 ± 16.7^c^	254.2 ± 14.8^b^	159.4 ± 10.3^a^	250.7 ± 16.0^b^	270.1 ± 18.6^b^
Folic acid	733.0 ± 22.3^c^	727.4 ± 23.1^c^	565.7 ± 13.7^b^	230.7 ± 0.1^a^	578.0 ± 35.7^b^	1,417 ± 24^d^
PABA	6.92 ± 0.50^c^	5.81 ± 0.26^b^	5.61 ± 0.10^ab^	5.30 ± 0.07^a^	6.53 ± 0.22^c^	19.19 ± 0.06^d^
α‐tocopherol	ND	ND	ND	ND	ND	ND
Retinol acetate	ND	ND	ND	ND	ND	ND
Total carotenoids (µg/100 g DW)	65.84 ± 0.79^d^	83.57 ± 3.12^e^	54.53 ± 0.81^b^	43.74 ± 1.98^a^	59.84 ± 2.64^c^	818.7 ± 25.3^f^

Note

Values with different superscripts within the same line are significantly different at *p* < .05 (*n* = 3).

Abbreviations: DW, dry weight; ND, not determined; PABA, p‐aminobenzoic acid.

* Ascorbic acid (mg/100 g DW).

### 3.2 Vitamins contents

Vitamin contents of powder samples are given in Table 2. Ascorbic acid content of unsieved powder from *D. glomerata* fruits was 59.7 mg/100 g DW, while thiamin, riboflavin, para‐amino benzoic acid, and folic acid contents were 12.2, 250.7, 6.5, and 578.0 µg/100 g DW, respectively. From a comparative point of view, ascorbic acid value found here was lower than that reported by Abdou Bouba et al. (2012) with mean value of 2.8 g/100 g DW. Acetate retinol and α‐tocopherol contents were not detected in all powder samples. Yet, Abdou Bouba et al. (2012) found α‐tocopherol content of 2.0 g/100 g DW. It must be recognized that production of plant powder is accompanied by physicochemical alteration induced by oxygen or heat (brought to plant during drying or grinding process) and can result in loss of the most sensitive vitamins. Indeed, vitamin A (retinol acetate) is sensitive to oxygen, temperature, and light (Loveday & Singh, 2008). During grinding operation, the particle temperature in the grinder surrounding can rise up to 90°C. Consequently, alteration of acetate retinol and α‐tocopherol may occur, thus inducing the reduction of their content in the powder extract. In addition, storage of powder affects the vitamin A stability. Hemery et al. (2018) reported a loss of 50% and 85% vitamin A in wheat powders stored at 25°C for three months, respectively, in polyethylene and paper bags (permeable to oxygen and moisture).

Looking to powder extracts obtained by CDSp, there was an increase in vitamin contents with decrease in particle size. From a comparative point of view between CDSp and ethanolic extraction, higher ascorbic acid, thiamin, and riboflavin contents were recorded in powder extracts obtained by CDSp, while ethanolic powder extract showed the highest contents in folic acid (1,417 µg/100 DW) and p‐aminobenzoic acid (19.1 µg/100 DW). Generally, thiamin, riboflavin, folic acid, and para‐amino benzoic acid contents were higher in the smallest particle size or intermediate granulometric fraction of powders obtained by CDSp. This could be explained by the fact that grinding followed by sieving process would induce the release of vitamins with increase of the powders specific surface area. Vitamins are synthesized in chloroplasts, mitochondria, and cytosol of plant cells (Smith, Croft, Moulin, & Webb, 2007); thus, their bioaccessibility was increased after the breakdown of plant cells during the grinding process. Yu and Kies (1993) also reported an increase in B vitamin bioaccessibility (niacin, pantothenic acid, and thiamine) in finer powder particle sizes. Nevertheless, there was often a loss of vitamins, attesting the deleterious impact of grinding process already discussed above.

### 3.3 Total carotenoid contents

Table 2 show the carotenoid contents of CDSp powder fractions, unsieved powder, and lyophilized ethanolic extract. Total carotenoid content of unsieved powder from *D. glomerata* fruits was 59.8 μg/100 g DW. Ethanolic extract presented a higher carotenoid content (818.7 μg/100 g DW) as compared to the powder fractions produced by CDSp. Indeed, ethanol extraction concentrated total carotenoids about 13 times than that of unsieved powder. Thus, carotenoids which are classified as a nonpolar compounds have a good solubility in ethanol, that is, a polar solvent. In addition, significant difference (*p* < .05) in total carotenoids contents was observed according to particle size. Powder fractions obtained by CDSp showed an increase in total carotenoid content. The smallest powder particles of 180–212 µm (83.57 ± 3.12 µg/100 g DW) showed the highest total carotenoid content; however, level in carotenoid content remained smaller as compared to that of ethanolic extract. Indeed, carotenoids are localized in chloroplasts and chromoplasts, which are surrounded by cell walls. Therefore, cell walls constitute physicochemical barriers which limits bioaccessibility of carotenoids. Thus, grinding process breaks the plant cell walls, facilitating nutrients' release and improvement of their bioavailability. Release of carotenoids depends on the level of rupture of plant tissues: The smallest particle size means that the highest specific surface area of particles, and more carotenoids are release on particle surface (Low, D'Arcy, & Gidley, 2015). Similar observations have been reported by Gong, Deng, Han, and Ning (2015) for carrot powders with an increase of about 50% in total carotenoid content when the particle size decrease from 240 to 40 μm. Nevertheless, a reduction in carotenoids content was observed below particle size of 180 μm. Generally, carotenoid contains a hydrocarbon with an unsaturated double bond or its oxygen derivatives, which are unstable and sensitive to oxygen, light, heat, and acid (Faure et al., 1999). Thus, alteration speed of carotenoids increases with increasing temperature, light exposure, and contact area with greater contact area inducing greater carotenoid lost. Then, surface area obviously increases for the smaller particle sizes, condition which provides a greater surface contact with environment and allow oxidation induced by light and oxygen (Ortiz, Ponrajan, Bonnet, Rocheford, & Ferruzzi, 2018). On the other hand, local temperature increases during grinding of plant matrix (Hu, Chen, & Ni, 2012) and could trigger isomerization or carotenoid alteration (Hwang, Stacewicz‐Sapuntzakis, & Bowen, 2012). Four types of chemical reactions can occur: rearrangement (isomerization), dehydration, oxidation, and cleavage (Mordi, 1993).

### 3.4 In vivo antioxidant activity

#### 3.4.1 Lipid peroxidation

**FIGURE 1:**
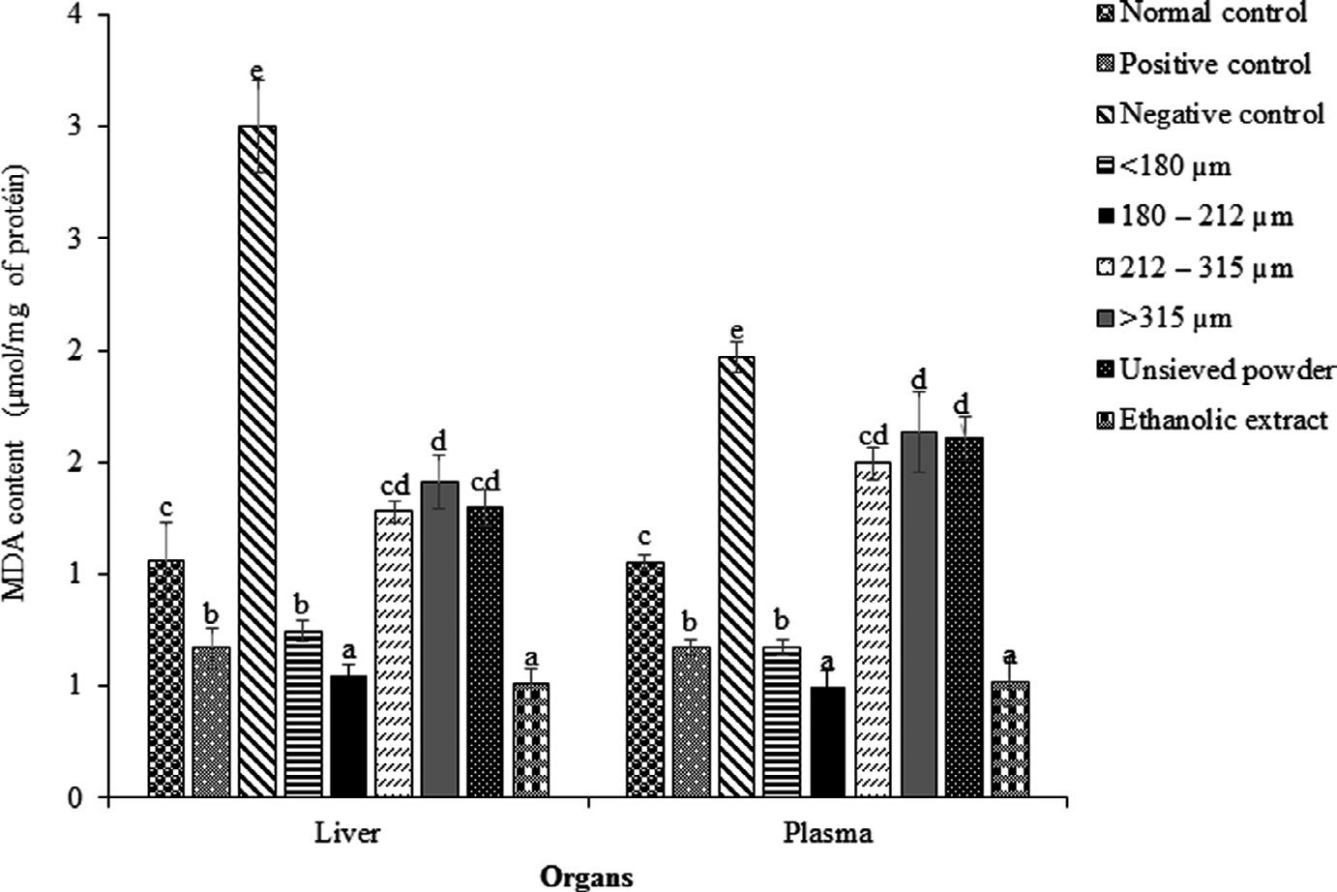
MDA content of liver and plasma of rats fed high‐fat diet and administered different powder fractions and ethanolic extract powder of *Dichrostachys glomerata*, and standard antioxidant ascorbic acid. Bars are means of three replicates, and error bars represent standard deviation. Within each organ, bars not having the same letter indicate significant difference at *p* < .05

Malondialdehyde is an indicator for lipid peroxidation of cell membranes. According to Figure 1, high‐fat diet‐induced significant increase in the plasma and liver MDA levels in untreated and high‐fat diet‐fed rat. An increase of 66% MDA was then observed when compared to MDA in organs of rats fed HFD (negative control) and that fed normal diet (normal control). Concomitant consummation of ascorbic acid (positive control) not only limits the increase of MDA in those organs, but also led to a significant decrease as compare to normal control. In fact, the MDA levels in organs of positive control rat fed ascorbic acid were significantly lower as compared to normal control rats. In conformity with previous studies by Ngatchic et al. (2013) and Pahane, Tatsadjeu, Bernard, and Njintang (2017), this result clearly points out that high‐fat diet is associated with oxidative stress as response to free radical production. Therefore, MDA appears as a pertinent indicator of failure of antioxidant defense mechanism of the organism. Worth mentioning is that MDA is produced by lipid oxidation in cell membrane (Thura, Salah, & Ithar, 2018). The mechanism is that hydroxyl radical (OH^●^) attacks polyunsaturated fatty acid, forming a carbon‐centered lipid radical. The radical rearranges to form a conjugate dienyl radical. This radical reacts with ambient oxygen (O_2_), forming a hydroperoxyl radical, which then abstracts a hydrogen from a neighboring lipid, forming a lipid peroxide and starting a chain reaction. This reaction continues until the supply of polyunsaturated fatty acid is exhausted, unless a termination reaction occurs.

Ascorbic acid appears here as a powerful antioxidant which not only stops peroxidation that may be induced by high‐fat diet but also reduced that was due to the normal diet. This also reveals that diet is also a source of oxidation which may impart the antioxidative system of the organism. The level of MDA in organs was comparable between normal groups, HFD groups administered ascorbic acid, and those administered *D. glomerata* powders and ethanol extract. This means that *D. glomerata* also played a vital role by inhibiting the oxidation that could have caused HFD. However, the effect of the powder varied with the fraction. While particle powder of 180–212 μm and ethanolic extract had the highest activity (lower MDA levels), the powder fraction <180 μm followed, and the powders 212–315 μm and ≥315 μm ended. The difference in the MDA activity is probably a consequence of the unequal repartition of antioxidant molecules during fractionation. For instance, it may be think that ascorbic acid is more concentrated in the fraction 180–212 μm than in the others, and this is reported above. But ascorbic acid is not the only antioxidant; total carotenoids and minerals were equally reported in relatively high quantities in this fraction. Our results equally highlighted the stronger inhibitory activity of the finest powders fraction as compared to coarser particles. Indeed, powders' finer particle (<180 μm and 180–212 μm) had previously been found to be richer in antioxidant compounds, especially in phenolic compounds (Deli, Baudelaire, et al., 2019), carotenoids and vitamins. For example, phenolic compounds have ability to neutralize free radicals, thus helping animal body to protect against oxidative stress (Wojdylo, Oszmianski, & Laskowski, 2009). Finally, ethanol which is generally used to prepare extract of high antioxidant activity seems to limit lipid peroxidation in a similar extend as our fraction size 180–212 μm. Fractionation has another advantage to ethanol extraction in the sense that it limits the use of solvent, and as such environmental and toxicity problems. This result opens new perspective for preparation of high antioxidant power drugs with eco‐friendly techniques.

#### 3.4.2 SOD and CAT activities

**FIGURE 2:**
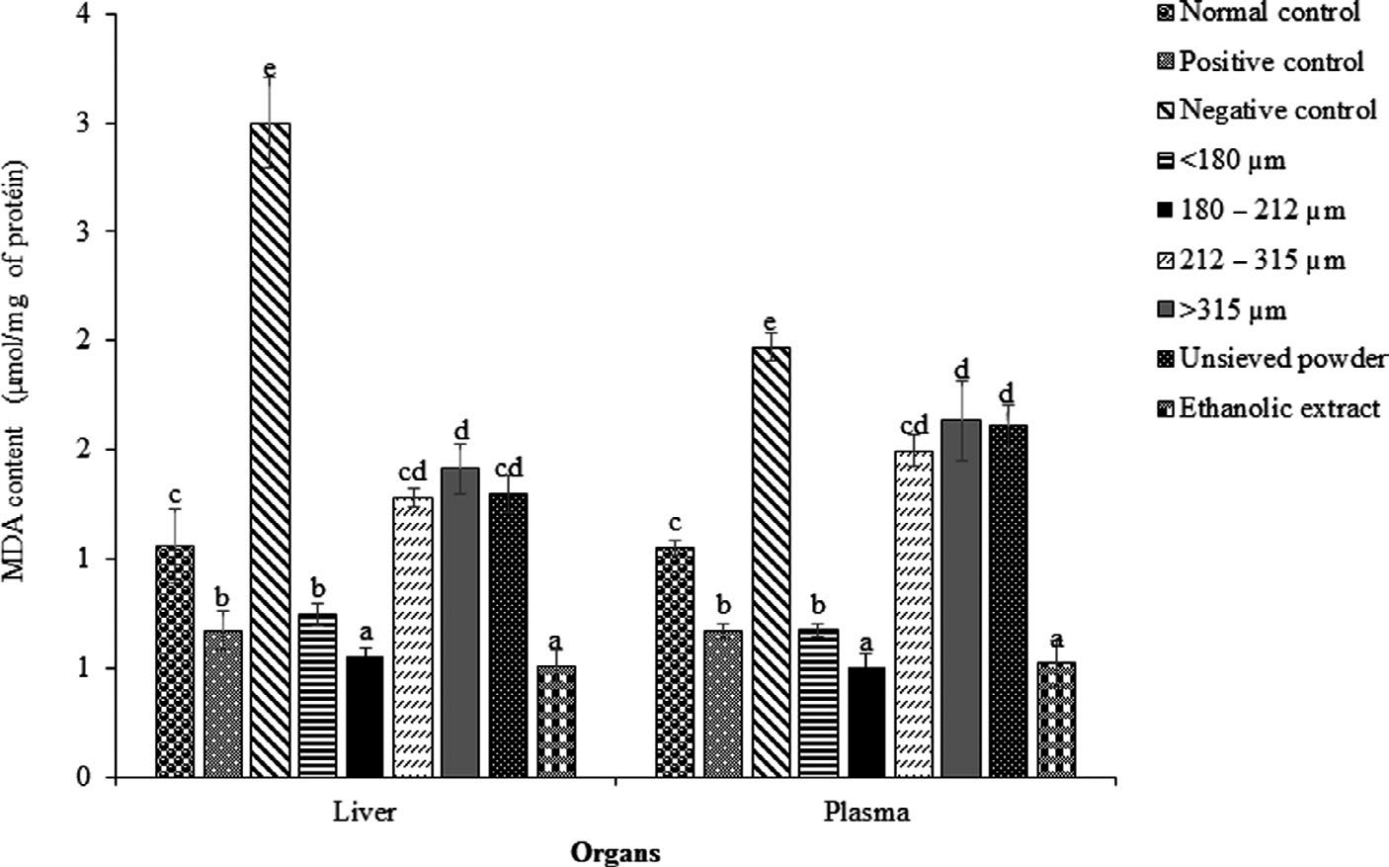
Effect of different fraction powders and ethanolic extract from *Dichrostachys glomerata*, and ascorbic acid on liver and plasma SOD activity in rat groups whose oxidative stress was induced by high‐fat diet. Bars are means of three replicates, and error bars represent standard deviation. Within each organ, bars not having the same letter indicate significant difference at *p* < .05

**FIGURE 3:**
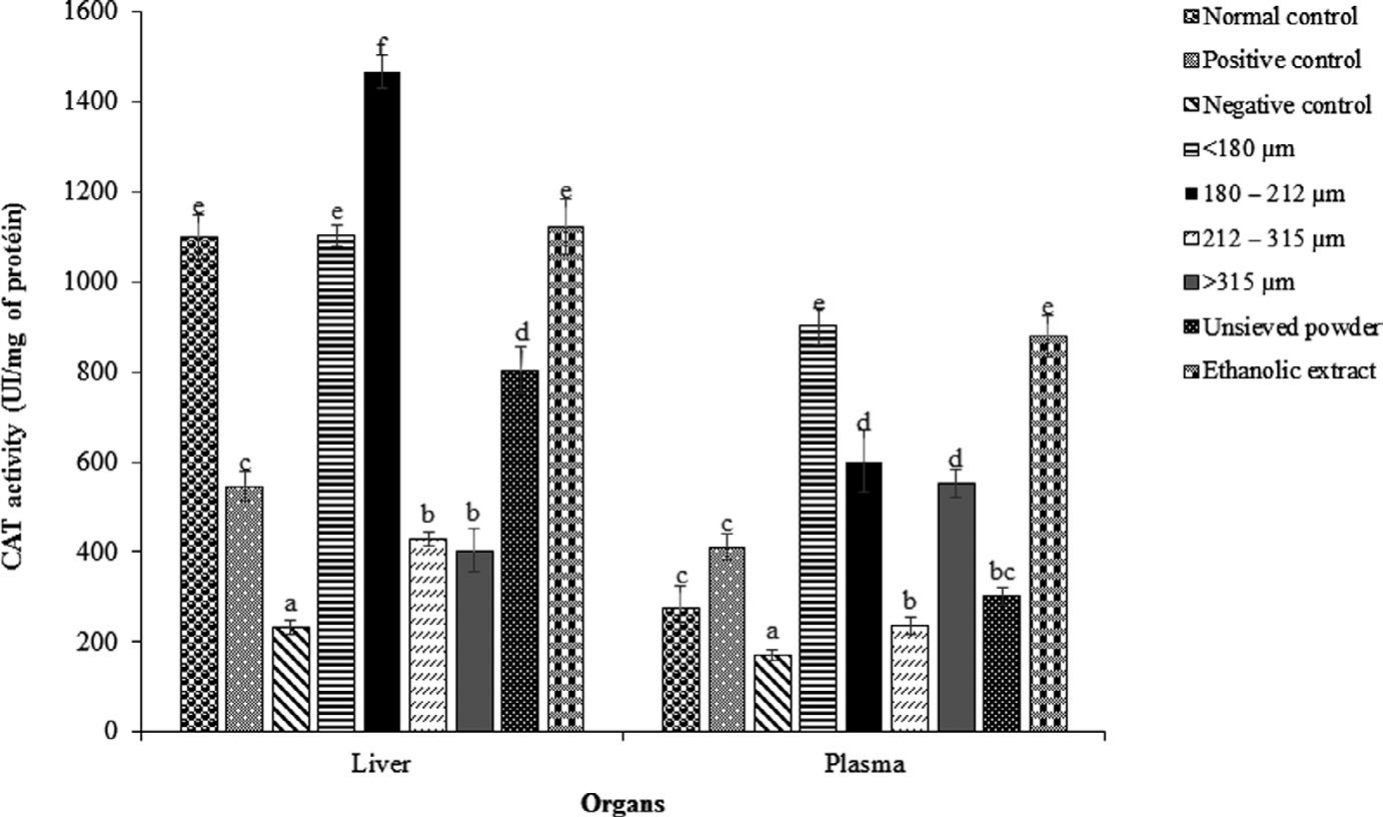
Effect of different fraction powders and ethanolic extract from *Dichrostachys glomerata*, and ascorbic acid on liver and plasma CAT activity in rat groups whose oxidative stress was induced by high‐fat diet. Bars are means of three replicates, and error bars represent standard deviation. Within each organ, bars not having the same letter indicate significant difference at *p* < .05

Liver and plasma SOD and CAT activities are shown in Figures 2 and 3. SOD and CAT are antioxidant enzymes involved in neutralizing free radicals. Recent studies report that their level diminished in oxidative stress conditions, in particular when fed HFD (Piao, Choi, Kwon, & Ha, 2017; Skowron et al., 2018). The present work equally reported that the treatment having the lowest SOD and CAT levels is rat group fed HFD without any treatment (negative control), while normal control rats had higher concentration. The lower activity of the first‐line antioxidant enzymes activities may result from inhibition by complexation of the enzymes. The decrease of liver and plasma CAT and SOD activities corroborates the previous results of MDA contents. By inhibiting the enzymes activity, the radical is then able to catalyze reactions. Thus, the tissues' MDA levels increase, while SOD and CAT activities decrease. Otherwise, SOD and CAT activities supposed to maintain the pro‐oxidant/antioxidant balance was not sufficient to control production of free radicals induced by high‐fat diet, and consequently oxidative reactions take place, and an increase in MDA levels constitutes a probably indicator. It can be also noted that in rats treated with ascorbic acid, powder fractions, unsieved powder, and ethanolic extracts of *D. glomerata*, a significant increase in SOD and CAT activities was observed as compared to that of negative control group. An administration of powder fractions, unsieved powder, and ethanolic extract of *D. glomerata* or vitamin C in rat groups subjected to oxidative stress probably entails an inhibition of free radical production. One plausible explanation is that the powder fractions from *D. glomerata* are powerful antioxidants which strongly scavenge most of the free radicals that accumulate in the body, thereby compensating the endogenous antioxidant potential in rats.

Looking to powder fractions, CAT and SOD activities were significantly (*p* < .05) affected by particle size. The highest plasma and liver CAT activity was observed in rat groups treated with powders of 180–212 μm. Comparatively to ethanol extract, plasma CAT and SOD activities of powders of 180–212 μm were equal, and interestedly greater to that obtained with vitamin C (positive control). This result could be explained by the level of antioxidant compounds in *D. glomerata* powders such as phenolic compounds, carotenoids, vitamins (vitamin C), and minerals. Phenolic antioxidants act by chelating metal ions, adsorbing and neutralizing free radicals, quenching singlet and triplet oxygen, or decomposing peroxides, and improving the antioxidant endogenous system, while minerals such as Zn and Cu act as cofactors of CAT and SOD activities. In addition, carotenoids are known to be very efficient physical and chemical quenchers of singlet oxygen (^1^O_2_), as well as potent scavengers of other reactive oxygen species (Cvetkovic, Fiedor, Fiedor, Wiśniewska‐Becker, & Markovic, 2013). The molecular mechanisms underlying these reactions are still not fully understood, especially in the context of the anti‐ and pro‐oxidant activity of carotenoids, which, although not synthesized by humans and animals, are also present in their blood and tissues, contributing to a number of biochemical processes (Fiedo & Burda, 2014). *Dichrostachys glomerata* powder is then a mixture of different chemical compounds endowed with antioxidant activity, and it would be difficult to attribute antioxidant effect to one or some active ingredients (Kurutas, 2016).

#### 3.4.3 Principal component analysis of micronutrients contents and in vivo antioxidant properties of *D. glomerata* powders

**FIGURE 4:**
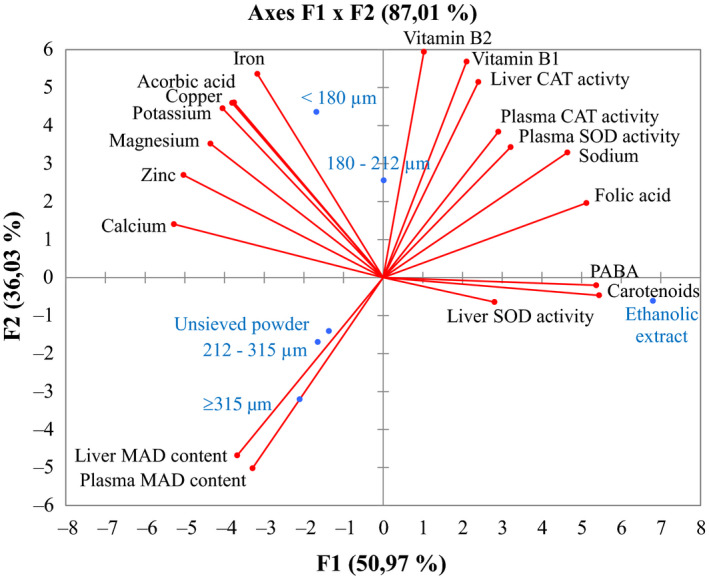
Projection of minerals, vitamins, carotenoids, in vivo antioxidant properties, and studied powder samples in factor plane F1‐F2 of principal component analysis

Figure 4 presents the variables on the correlation circle and the mapping of individuals (drug treatments) on the principal components axes PC1 and PC2, which express 87% of the total variability. Globally, the variables were organized into three main categories: those at the upper left which contributed negatively to PC1 and positively to PC2, the variables at the upper right which contributed positively to both PC1 and PC2, and finally those at the lower left which is composed of MDA levels, and those on the right PC1 axis composed of carotenoids and PABA, or liver SOD activity. In general, vitamin C, iron, zinc, and copper contents were positively correlated with each other and higher in the finest powders of 180 and 180–212 µm as compared to powders with large particle size. The large particle powders (212–315, ≥315 μm) and unsieved powder were lesser rich in minerals and vitamins and also had high MDA level contents. The MDA levels were negatively correlated with vitamins B1 and B2, Cat and SOD activities, folic acid, and sodium contents. This figure clearly revealed that minerals and vitamins jointly contributed to the increase in CAT and SOD activities and decrease MDA in finer powder (<180 and 180–212 µm) fractions, the coarser powder fractions (unsieved powder, 212–315, ≥315 μm) are definitely lower in vitamins, while the antioxidant power of ethanol fraction derived particularly from its high level in carotenoids and PABA. Worth important to notice was the correlation between CAT and SOD activities in liver and plasma in the exception of liver SOD activity which was not correlated with the CAT liver activity. Most of the CAT and SOD activities seem to be highly correlated with the vitamins B1 and B2 and folic acid, while the liver SOD was correlated with the total carotenoids. Research in first‐line antioxidant enzyme has been the subject of many research. Notably, SOD enzyme exists actually as a nutritional supplement from plants such watermelon. This result claimed the potential role of *D. glomerata* on increasing the antioxidant enzymes activity of SOD and CAT.

## 4 CONCLUSION

In this study, the grinding process followed by controlled differential sieving process (CDSp) was compared to ethanolic extraction from *D. glomerata* fruits. According to the results, the micronutrients contents and in vivo antioxidant properties were greatly affected by the particle size of *D. glomerata* fruits powder. A decrease in particle size is associated with an increase in the levels of minerals, total carotenoids, and vitamins contents. The powders finest particle <180 µm and 180–212 μm were found to possess higher contents of minerals and vitamins and also to exhibit higher preventive antioxidant activity as compared to coarser particles which were poor in vitamins, but contain substantial amounts of minerals. The ethanolic extract was essentially rich in total carotenoids. Overall, grinding and CDS process is helpful for improving the minerals, total carotenoids, and vitamin contents alongside the antioxidant properties of *D. glomerata* powder.

## CONFLICT OF INTEREST

The authors declare no conflict of interest.

## ETHICAL APPROVAL

Human/animal testing is unnecessary in this study. Human subject is not involved in this study. Patients are also not involved in this study.
